# Liver Transplantation for Colorectal Liver Metastases: Current Management and Future Perspectives

**DOI:** 10.3390/ijms22063093

**Published:** 2021-03-18

**Authors:** Serban Puia-Negulescu, Fanny Lebossé, Jean-Yves Mabrut, Xavier Muller, Guillaume Rossignol, Teresa Antonini, Domitille Erard, Sylvie Radenne, Marielle Guillet, Jean-Christophe Souquet, Kayvan Mohkam, Mickael Lesurtel

**Affiliations:** 1 Department of Digestive Surgery and Liver Transplantation, Croix Rousse University Hospital, University of Lyon I, 69004 Lyon, France; puiaserban@gmail.com (S.P.-N.); jean-yves.mabrut@chu-lyon.fr (J.-Y.M.); xmuller.ucl@gmail.com (X.M.); kayvan.mohkam01@chu-lyon.fr (K.M.); 2 Department of Hepatology, Croix Rousse University Hospital, University of Lyon I, 69004 Lyon, France; fanny.lebosse@chu-lyon.fr (F.L.); teresa.antonini@chu-lyon.fr (T.A.); domitille.erard@chu-lyon.fr (D.E.); sylvie.radenne@chu-lyon.fr (S.R.); 3 Cancer Research Center of Lyon, INSERM U1052, 69008 Lyon, France; 4 Department of Pediatric Surgery, Hôpital Femme Mère Enfant, Hospices Civils de Lyon, University of Lyon I, 69500 Lyon, France; guillaume.rossignol@chu-lyon.fr; 5 Department of Hepatogastroenterology, Croix Rousse University Hospital, University of Lyon I, 69004 Lyon, France; marielle.guillet@chu-lyon.fr (M.G.); jean-christophe.souquet@chu-lyon.fr (J.-C.S.)

**Keywords:** liver transplantation, colorectal cancer, colorectal liver metastases, organ donation, recurrence

## Abstract

Patients with nonresectable liver metastases from colorectal cancer have few therapeutic options and a dismal prognosis. Although liver transplantation for this indication has historically a poor reputation, recent advances in the field of chemotherapy and immunosuppression have paved the way to revisit the concept. New data have shown promising results that need to be validated in several ongoing clinical trials. Since liver grafts represent a scarce resource, several new tools are being explored to expand the donor pool for this indication. The purpose of this review is to present all current available data and perspectives about liver transplantation for nonresectable liver metastases from colorectal cancer.

## 1. Introduction

Colorectal cancer is the most frequent cancer of the digestive tract, being third in order of frequency and fourth in the order of mortality amongst all cancer patients [1]. More than half of all patients with colorectal cancer (CRC) will develop liver metastases during their lifetime [2]. The only hope for a cure is a hepatic resection combined with perioperative chemotherapy, but less than half of all patients are or will become resectable after chemotherapy [3]. Furthermore, tumor-free margin hepatectomy potentially leaves behind undetected metastases in the remnant liver, which will eventually be responsible for recurrence in more than 50% of cases [4]. Among those patients, only a minority will undergo a re-hepatectomy [5]. For nonresectable liver metastases, survival is poor with only 10% of patients being alive after 5 years even with the best chemotherapy regimens [6]. Liver transplantation (LT) offers the theoretical advantage of a real R0 resection, removing all potentially undetected metastases. From a practical point of view, in the current era of liver graft shortages, an ethical question arises of whether or not such a precious resource should be used for a poorly explored marginal indication such as nonresectable colorectal liver metastases (CRLMs).

## 2. Historical Results

The first considerable experience with LT for nonresectable CRLM was reported by the University of Vienna in 1991 [7]. The twenty-five patients who underwent LT achieved a disappointing 5-year survival rate of only 12%. Almost one-third of patients died by postoperative day 30 and two-thirds of them recurred by the end of the study. The University of Cincinnati published in the same period the results of a large cohort of LTs [8]. Of 637 patients who were transplanted, only 41 underwent LT for metastatic liver lesions, of which 10 were nonresectable CRLM (including two patients with liver failure after chemotherapy). There were no specific data about patients with nonresectable CRLM, but patients with metastatic disease had a survival rate of only 21% after 5 years. The 30-day mortality rate was 11%, and 70% of patients recurred, confirming the disappointing results of the Vienna group.

The European Liver Transplantation Registry (ELTR) published the results of LT for nonresectable CRLM performed between 1977 and 1995 [9]. The majority of patients belonged to the series of the Vienna group and the overall results were similar, with a 5-year survival rate of 18%. The authors emphasized that graft loss was not related to tumor recurrence in 44% of patients, reflecting issues with perioperative management and the immunosuppression regimens used at that time.

In the context of graft shortages and poor oncological results, LT for nonresectable CRLM failed to establish itself as a justified indication, and the concept was therefore considered as an absolute contraindication for a long period of time.

## 3. Why Reconsider LT for Nonresectable CRLM?

First, important progress has been made in the field of chemotherapy for metastatic CRC since the publication of the abovementioned studies, which used no or conventional chemotherapy. In the 1990s, studies using 5-FU and leucovorin for CRLM showed a 1-year overall survival (OS) and response rate of around 30% [10,11]. A key event was the introduction in the early 2000s of irinotecan and oxaliplatin as a combination therapy that allowed for an improved response rate with a median survival rate of approximately 2 years. The FOLFOX and FOLFIRI regimens achieved a response rate of 50% [12], which even increased to 60% with the FOLFIRINOX triplet [13]. The 2000s also brought biological agents into the treatment of CRC metastases. Adding anti-angiogenic therapy to the classical chemotherapy doublet induced a 2-fold increase in the response rate [14]. The addition of anti-epidermal growth factor receptors (EGFRs) improved response and survival rates compared with conventional chemotherapy [15,16]. The discovery of intra-tumoral mutations of the RAS and BRAF genes refined the criteria for the administration of biological therapy. Indeed, several studies clearly showed that tumors bearing these mutations did not respond well to anti-EGFR therapy [17,18]. The newly approved chemotherapy agents for refractory CRLM (multiple kinase inhibitors and TAS-102) may open new perspectives compared with best supportive care [19,20]. Overall, progress in the chemotherapy armamentarium allowed for better control of the metastatic disease.

Second, the efficacity of newly introduced immunosuppression agents significantly improved survival after liver transplantation. In a recent retrospective study comprising 111,568 patients transplanted from 1987 to 2017, the 1-year survival rate increased from 66% in 1986 to 92% in 2015 [21]. The addition of calcineurin inhibitors (CNIs) to the classical immunosuppression protocols in the 1980s was a breakthrough [22]. Tacrolimus is nowadays the preferred CNI because it enables a low rejection rate, less graft loss, and fewer side effects compared with cyclosporine [23,24]. The combination of mycophenolate mofetil (MMF), the most commonly used antimetabolite, with CNI permits the administration of a lower dose of CNI. This regimen is associated with a lower toxicity profile than CNI monotherapy, especially regarding renal function [25]. In the late 1990s, mTOR inhibitors were introduced in the field of transplantation and are generally used in combination with MMF and tacrolimus [26]. While evidence in the literature is little, mTOR inhibitors may offer an anti-tumoral effect, thus being an attractive agent for patients receiving LT for malignant diseases [27]. Additionally introduced in the late 1990s, basiliximab is an anti-CD25 monoclonal antibody that offers decreased rates of acute cellular rejection and preserves renal function [28]. A standard immunosuppression protocol proposed by the team of the Oslo University Hospital for LT for nonresectable CRLM consists of induction with basiliximab, tacrolimus for the first 4–6 weeks, and then conversion to sirolimus (an mTOR inhibitor). Glucocorticoids and MMF are administered from day one and glucocorticoids are tapered to zero during the first 3 to 6 months [29].

Finally, ethical considerations are important for this indication since liver grafts have become a scarce resource. Even for validated indications for LT, the waiting time until transplantation is long. It varies throughout the globe: less than 4 months in Spain, 4 to 5 months in the United Kingdom, and 11.3 months in the United States of America [9,30]. Scandiatransplant, a collaboration of all organ transplant centers in the Nordic countries (Norway, Sweden, Finland, Denmark, and Iceland), defies the standard waiting time with only 39 days on the waiting list [31]. This particular situation allows those transplantation teams to explore other indications for LT, notably nonresectable CRLM [32].

## 4. New Results

In 2013, the LT team at Oslo University Hospital published the results of the Secondary Cancer (SECA) I study, 21 patients who underwent liver transplantation for nonresectable CRLM between 2006 and 2011 [32]. The main inclusion criteria were a radical resection of the primary tumor, a minimum of 6 weeks of chemotherapy, and the absence of extrahepatic disease. All patients underwent an exploratory laparotomy with lymphadenectomy of the hepato-duodenal ligament. Patients with positive nodes were not transplanted. After LT, no adjuvant chemotherapy was delivered, and the immunosuppressive protocol included the mTOR inhibitor sirolimus from the first postoperative day. The 5-year OS was 60%, but most patients (19/21) recurred after a median of only 6 months. Four factors impacted survival negatively: tumors larger than 5.5 cm, carcinoembryonic antigen (CEA) over 80 µg/L, surgery of the CRC primary less than 2 years before the LT, and progression of metastases at the time of LT. These factors constitute the Oslo score, with one point given for each negative factor present. Unique of its kind at the time, it paved the way for a renewed interest in LT for nonresectable CRLM by showing that long-term survival was possible in the majority of these patients.

The question of long-term survival without disease after LT remained open until the 2015 retrospective study of the Compagnons Hépato-Biliaires [33]. This multicentric study included 12 patients who were transplanted between 1995 and 2015. No patient had progression of the disease at the time of LT and the procedure took place more than 1 year after excision of the primary. Half of the patients received a planned surgery and half underwent a “compassionate” LT. The 5-year OS was 50%, lower than in the SECA I study. Despite the highly heterogenous group of transplanted patients, 5/12 were still alive with no recurrence at 7, 43, 47, 48, and 108 months after LT, all belonging to the scheduled procedure group.

The relative abundance of liver grafts in Norway permitted the Oslo group to further explore the matter of LT for patients with nonresectable CRLM [29]. Their recent prospective study reported the results of 15 patients from the ongoing four-arm SECA II trial (NCT01479608) transplanted between 2012 and 2016 (mostly in arm C: LT in nonresectable patients with synchronous disease). More refined criteria were applied. Besides the absence of extrahepatic disease and the radical resection of the primary, the other inclusion criteria were at least one line of chemotherapy with at least a 10% response rate using RECIST 1.1 criteria, no lesions larger than 10 cm, and a 1-year delay between diagnosis of the primary and listing for LT. The 5-year OS was 83%. The median disease-free survival (DFS) was 13.7 months, including four patients without any signs of recurrence at 2 years.

The Oslo group’s latest publication reported the preliminary results of arm D of the ongoing SECA II trial [34]. This fourth arm included 10 patients with aggressive liver synchronous metastases with potentially pulmonary metastases. The authors used extended criteria liver grafts including having a steatosis of over 60%, coming from donation after circulatory death donors, donors over 70 years of age, donors with previous hepatitis B infection, and donors with previous malignancy. Median DFS and OS were 4 and 18 months, respectively. In addition to enhancing the limits of LT for nonresectable CRLM, the study showed that there was no evidence of transmission of the initial donor malignancy or the hepatitis B infection. All the patients had a functional liver at the time of death.

In summary, the recent experience showed that acceptable results could be obtained using strict selection criteria. In other words, the best survival was obtained in patients with metastases well controlled by chemotherapy and limited to the liver.

## 5. Validation of LT as a Standard Treatment Option for Patients with Nonresectable CRLM

To be validated as a useful indication for nonresectable CRLM, LT should offer an advantage over chemotherapy alone.

The survival rate of nonresectable CRLM treated with chemotherapy only reaches 10% at 5 years. The Oslo group compared the SECA I group of patients to a similar group of patients receiving chemotherapy as a stand-alone treatment at their institution in the Nordic VII trial [35]. Both groups had similar tumor characteristics. The 5-year OS was 56% in the SECA I group versus 9% in the Nordic VII group. DFS was, however, similar (10 vs. 8 months). Even the best 5-year OS of 21 patients from the Nordic VII group reached only 19%.

The outcomes of these studies support that LT could be an acceptable oncological treatment of nonresectable CRLM with selected patients. Additional support should come from three ongoing trials comparing LT to chemotherapy. TRANSMET (Liver Transplantation in Patients with Unresectable Colorectal Liver Metastases Treated by Chemotherapy- NCT02597348) is a French multicentric randomized controlled trial comparing LT after neoadjuvant chemotherapy to chemotherapy as a stand-alone treatment. The included patients have metastases limited to the liver, show no signs of progression under chemotherapy after 3 months, and show no signs of recurrence of the primary. The protocol mandates that chemotherapy should be resumed after LT. The COLT (Improving Outcome of Selected Patients with Non-resectable Hepatic Metastases from Colo-rectal Cancer with Liver Transplantation) study is an Italian multicentric non-randomized study comparing LT after neoadjuvant chemotherapy to chemotherapy in a cohort of patients with the same tumor characteristics (NCT03803436). The inclusion criteria are similar to the French trial and insist on including patients with early, non-aggressive primary tumors. The SECA III trial is a monocentric randomized controlled trial from the Oslo group comparing LT to alternative therapies in patients who progressed during the first line of chemotherapy (NCT03494946). The metastatic disease has to be limited to the liver, except for resectable pulmonary metastases with a maximum diameter of 15 mm.

## 6. Patterns of Recurrence after LT

Although having an acceptable 5-year OS, patients with nonresectable CRLM who underwent LT tend to have early recurrences. The SECA I group of patients was analyzed regarding the patterns of recurrence in a specific study [36]. Two patterns were evident. In one pattern, pulmonary metastases were the first site of recurrence (68%). They occurred early, 4 months after LT, had an indolent course, and most were amenable to curative resection. Thus, the 5-year OS of this group reached 72%. In the second pattern, hepatic metastases occurred as multi-site recurrences while the liver was never the first site. These patients had the worst survival rate. An interesting finding was that retrospective analysis of CT scans showed that 33% of those patients had undetected pulmonary metastases. Despite metastatic lung disease, those patients were among the ones with the longest survival in the SECA I study.

This pattern of recurrence was confirmed in the SECA II study with a 70% pulmonary recurrence rate, mostly being slow-growing and resectable [29].

These initial findings were in contrast with the Compagnons Hépato-Biliaires study and the arm D study of the SECA II trial [33,34]. These two studies showed the lungs as the most common site of recurrence, but these lesions were not amenable to curative treatment. However, the Compagnons Hépato-Biliaires study included a heterogeneous group of patients, half of whom underwent “compassionate” LT for aggressive colorectal cancer. Similarly, the SECA II arm D study included patients who had or were resected for pulmonary metastases, emphasizing again the aggressive nature of their disease.

## 7. Selection of Patients and Prognostic Factors

The Fong clinical risk score (FCRS) is one of the oldest scores evaluating OS in patients who undergo liver resections for CRLM [37]. It allocates one point for each of the following prognostic factors: synchronous disease (metastases detected less than 1 year from the diagnosis of the primary), more than one liver lesion, size of the largest tumor more than 5 cm, CEA levels above 200 μg/L, and an N+ primary tumor. The Oslo score specifically evaluates the OS of LT patients, giving one point for each present prognostic factor: tumors larger than 5.5 cm, CEA over 80 μg/L, surgery of the primary less than 2 years before the LT, and progression of metastases at the time of LT [32]. Additionally, the Oslo group compared metabolic tumor volume (MTV) on PET-CT, the FCRS, and the Oslo score in patients from the SECA I and SECA II studies after a long follow-up of 85 months [38]. An MTV level below 70 cm^3^ translated to low FCRS and Oslo scores, regardless of the T and N stages of the primary tumor, K-RAS mutation status, sidedness of the primary tumor (right vs. left), or time from the surgery of the primary and LT. Specifically for the FCRS score, a value of 0–2 offered a significant survival advantage over a score of 3–5. An Oslo score of 0–2 also offered a significant survival advantage over a score of 3–4. Although not statistically significant, K-RAS wild-type tumors led to better survival than their mutated counterparts (73 months vs. 40 months). Compared with right-sided tumors, left-sided ones had a better DFS and 5-year OS (4 months vs. 13 months and 0% vs. 66%, respectively). The best survival rate was obtained using an FCRS of 0–2, but this criterion would have been extremely restrictive, and fewer than half of the patients would have been transplanted.

The only study that included BRAF-mutated tumors was the SECA II arm D study [34]. Two patients were included. Their survival rate was very different, and no robust conclusion could be drawn: the first patient had a survival time of only 6 months and the second is still alive at 26 months after LT.

## 8. Ongoing Studies

There are currently eight ongoing studies focusing on the role of LT in patients with nonresectable CRLM. They explore various aspects of this complex topic: LT vs. chemotherapy, liver resection vs. LT for resectable CRLM, and assessment of new means to expand the donor pool of liver grafts. The main features of these trials are summarized in Table 1.

Molecular prognostic factors are currently gaining wide acceptance in selecting therapies for patients with CRLM. Half of the ongoing studies include molecular tumor markers in their inclusion criteria. The TRANSMET and the Toronto trial exclude patients with BRAF-mutated tumors. The SOULMATE (The Swedish Study of Liver Transplantation for Non-resectable Colorectal Cancer Metastases- NCT04161092) study includes only patients with BRAF wild-type tumors with microsatellite stability. Finally, the COLT study includes only patients with RAS and BRAF wild-type tumors with microsatellite stability.

## 9. New Concepts to Expand the Donor Pool

Because liver grafts are a scarce resource, LT is only offered to patients with validated indications in most countries. Expanding the donor pool of liver grafts could grant access to LT for patients presenting with marginal indications, such as nonresectable CRLM, for which LT is proposed to patients only in the setting of clinical trials.

Using extended criteria donors (ECDs) could be an interesting solution. The Oslo team showed that this option was safe in patients with CRLM [34], although survival was often associated with cancer recurrence. All patients who died from recurrence at the end of the study had a functioning graft at the time of death. Only one patient had to be retransplanted, initially receiving a graft with 80% steatosis and developing post LT primary non-function. Indeed, recipients with nonresectable CRLM do not suffer from portal hypertension nor liver insufficiency and may therefore tolerate better those marginal grafts. A prospective randomized trial from Sweden comparing LT with ECD vs. best alternative care for nonresectable CRLM will soon start recruiting patients (NCT04161092).

A novel concept was introduced in 2015 as a hybrid of the auxiliary LT and the ALPPS procedure [39]. The Resection and Partial Liver segment 2 and 3 transplantation with Delayed total hepatectomy (RAPID) protocol proposes a two-stage hepatectomy. The first step consists of segment 1–3 resection and transplantation of a left lateral (segments 2 and 3) graft. Pressure in the main portal trunk is monitored to have a target pressure of less than 20 mmHg to the small liver graft after the ligation of the right portal vein of the native liver. If the pressure is higher than 20 mmHg, a flow modulation is recommended including either ligation of the splenic artery, calibration of the portal vein, or portocaval shunting between the proximal right portal vein and the inferior vena cava. After the donor graft reaches at least 0.8% of the recipient’s body weight or 35%–40% of standardized total liver volume, resection of the remnant right native liver is performed. The advantage of the RAPID concept is that it does not reduce the liver donor pool. The technique uses a left lateral graft that would have had insufficient volume for a classical liver transplantation.

A prospective study is currently recruiting patients at the Oslo University Hospital (NCT02215889). The aim is to analyze the feasibility of the RAPID procedure by measuring the percentage of patients who undergo the second stage within 4 weeks after the first hepatectomy.

An ongoing bicentric prospective study from Germany extended the RAPID concept by using living donors for the segments 2–3 graft (NCT03488953).

The concept of using standard living donors is being assessed in another ongoing prospective trial from the Toronto group (NCT02864485). Using living donors has several advantages. First, there is a low perioperative risk for the donor, as only the left lateral segment is being procured. Second, being an elective surgery, living donor LT allows for more precise timing regarding the oncological sequence of treatment.

## 10. Conclusions

Although recent results of LT for nonresectable CRLM are encouraging compared with the historical experience that has banned it for a long period, current data are based mostly on small, monocentric, and heterogeneous studies. In the era of organ shortages, acceptable evidence is needed before including this option in the armamentarium of surgeons treating patients with nonresectable CRLM. Several ongoing trials may provide the evidence needed to validate such an indication.

Heterogenous access to LT among different parts of the world is another issue. Even after validating LT as a solid indication for nonresectable CRLM, only a minority of patients will be transplanted. This will impose a serious ethical burden, as the difference in survival is huge between patients receiving only chemotherapy and patients receiving LT for the same tumor profile.

The selection of patients is key to guarantee the maximum benefit of LT for this indication. As the surgical oncologist Dr. Blake Cady stated: “biology is king, selection is queen, technical maneuvers are the prince and the princess; occasionally, the prince or princess tries to usurp the throne; they almost always fail to overcome the powerful forces of the king and queen”.

## Figures and Tables

**Table 1:** Ongoing trials on liver transplantation for colorectal liver metastases (CRLMs).

Name, NCT Number and Location	Description	Inclusion Criteria	Primary Endpoint
TRANSMET NCT02597348 France	A multicentric randomized trial comparing 5-year survival of chemotherapy followed by LT vs. chemotherapy alone in patients with confirmed nonresectable liver-only colorectal metastases, well-controlled by chemotherapy	- more than 3 months of tumor control on chemotherapy - BRAF wild-type tumors - 2 or fewer lines of chemotherapy - no signs of extrahepatic disease/local recurrence of primary	5-year OS
SECA II NCT01479608 Oslo, Norway	A monocentric prospective 4-arm trial	- at least 6 weeks of chemotherapy	10-year OS
	ARM A: randomized controlled trial LT vs. surgical resection	For ARM A: 6 or more resectable liver lesions	
	ARM B: prospective study on liver transplantation for nonresectable CRLM in patients with metachronous disease.	For ARM B: - primary tumor pN0 - CEA less than 100 μg/L at the time of primary diagnosis - before the start of first-line chemotherapy, no lesion more than 10 cm, no more than 20 lesions, and at least 10% response after the first line of chemotherapy	
	ARM C: prospective study on liver transplantation for nonresectable CRLM in patients with the synchronous disease.	For ARM C: - at least 1 line of chemotherapy - before the start of the second or third lines of chemotherapy, no lesion more than 10 cm and no more than 20 lesions - at least 10% response after the second or third lines of chemotherapy - 2 years or more between the diagnosis of primary and listing for LT	
	ARM D: prospective study on liver transplantation for nonresectable CRLM in patients with the synchronous disease using extended criteria donors	For ARM D: - resectable or previously resected pulmonary lesions	
SECA III NCT03494946 Oslo, Norway	A monocentric randomized trial comparing the overall survival of patients with nonresectable CRLM receiving LT vs. other treatment that may include further chemotherapy, TACE, SIRT, or other available treatment options.	- no signs of extrahepatic disease, except resectable lung metastases (max 15 mm) - progressive disease or intolerance to first-line chemotherapy - Oslo score of less than 3 - lesion smaller than 10 cm	2-year OS
Rapid trial NCT02215889 Oslo, Norway	A clinical trial to evaluate the benefit and efficacy of liver resection and partial liver segment 2/3 transplantation with delayed total hepatectomy as a treatment for selected patients with nonresectable CRLM	- at least 8 weeks of chemotherapy - no signs of extrahepatic metastatic disease, except patients may have 1–3 resectable lung lesions all <15 mm.	% of transplanted patients receiving second stage hepatectomy within 4 weeks of segment 2–3 transplantation
LIVERT(W)OHEAL NCT03488953 Germany Jena and Tubingen	A bicentric clinical trial to evaluate the benefit and efficacy of liver resection and partial liver segment 2–3 transplantation with delayed total hepatectomy as a treatment for selected patients with nonresectable liver metastases from colorectal carcinoma using living donors	- nonresectable colorectal liver metastases without extrahepatic tumor burden, except resectable pulmonary metastases - stable disease or regression after at least eight weeks of systemic chemotherapy	3-year OS after the second hepatectomy
Toronto study NCT02864485 Toronto, Canada	A monocentric study to evaluate the results of live donor liver transplantation to selected patients with nonresectable metastases CRLM	- ≤T4a primary tumor - the interval between the resection of primary to transplant is ≥6 months - no major vascular invasion liver metastases - systemic chemotherapy for ≥3 months - stable or decreasing CEA values - BRAF wild-type tumors	5-year OS 5-year DFS
SOULMATE study NCT04161092 Sweden Gothenburg and Stockholm	A randomized controlled bicentric study evaluating if liver transplantation with liver grafts from extended criteria donors not utilized for approved indications increases overall survival in patients with nonresectable isolated CRLM, in comparison with best alternative care	- at least 2 months of chemotherapy with no progression - at least 1 year from the diagnosis of primary and the inclusion in the study - liver metastases less than 10 cm - BRAF wild-type tumors - MSS tumors	5-year OS
COLT study NCT03803436 Italy	A multicenter, non-randomized, prospective study assessing the efficacy of liver transplantation in liver only CRLM, compared with a matched cohort of patients bearing the same tumor characteristics, collected during the same period and included in phase III Italian randomized controlled trial on triplet chemotherapy+ anti-EGFR	- primary tumor as pT1-3, pN0, or pN1 - RAS and BRAF wild-type and MSS - objective response to first-line treatment, with a sustained response for at least 4 months, OR disease control during second-line treatment for at least 4 months. - a maximum of 2 prior chemotherapy treatment lines - CEA < 50 ng/mL - extra-peritoneal rectal cancer	5-year OS

CRLM: colorectal liver metastases, LT: liver transplantation, OS: overall survival, DFS: disease-free survival, CEA: carcinoembryonic antigen, TACE: transarterial chemoembolization, SIRT: selective internal radiation therapy, MSS: microsatellite stable.

## Data Availability

The data presented in this study are openly available (see references).
